# *SLCO2B1* genetic polymorphisms in a Korean population: pyrosequencing analyses and comprehensive comparison with other populations

**DOI:** 10.1007/s11033-013-2502-x

**Published:** 2013-05-11

**Authors:** Kyoung-Ah Kim, Hyun-Jin Joo, Hae-Mi Lee, Ji-Young Park

**Affiliations:** Department of Clinical Pharmacology and Toxicology, Anam Hospital, Korea University College of Medicine, 126-1, Anam-dong 5-ga, Sungbuk-gu, Seoul 136-705 Korea

**Keywords:** *SLCO2B1*, OATP2B1, Pyrosequencing, Pharmacogenetics, Koreans, Ethnic difference

## Abstract

SLCO2B1, also known as OATP2B1 (Organic Anion Transporter) or OATP-B or SLC21A9, is an organic anion uptake transporter that is encoded by the *SLCO2B1* gene. In this study we assessed the frequencies of *SLCO2B1* polymorphisms in a Korean population using newly developed pyrosequencing methods and compared their frequencies with those in other ethnic groups. We developed pyrosequencing methods to identify the following six *SLCO2B1* non-synonymous polymorphisms: c.1175C > T (rs1621378), c.1457C > T (rs2306168), c.43C > T (rs56837383), c.935G > A (rs12422149), c.601G > A (rs35199625) and c.644A > T (rs72559740). The allele frequencies of these polymorphisms were analyzed in 227 Korean subjects. The allele frequencies of *SLCO2B1* polymorphisms in the population tested were as follows: 0.0 for c.1175C > T, c.43C > T and c.644A > T; 0.2687 for c.1457C > T; 0.4273 for c.935G > A; and 0.0727 for c. 601G > A. Even though the allele frequencies of the c.1175C > T and c.1457C > T polymorphisms were comparable to those in Japanese subjects, the frequencies in this Korean population differed from those in other ethnic groups. The developed pyrosequencing methods are rapid and reliable for detecting non-synonymous *SLCO2B1* polymorphisms. Large ethnic differences in the frequency of *SLCO2B1* genetic polymorphisms were noted among ethnic groups. The *SLCO2B1* polymorphisms at c.1175C > T, c.43C > T and c.644A > T were not found in the Korean population while c.1457C > T, c.935G > A and c.601G > A exhibited mostly higher frequencies in Koreans compared with Finnish, Caucasian and African-American populations.

## Introduction

It has recently been recognized that drug pharmacokinetics, efficacy and toxicity are related to the role of drug transporters [1]. Solute Carrier Organic Anion Transporter 2B1 (SLCO2B1), also known as SLC21A9, OATP2B1 and OATP-B, encodes a member of the organic anion transporting polypeptide family of membrane proteins. The SLCO2B1 transporter is expressed at the sinusoidal membrane of hepatocytes in the liver, but also in other tissues including the intestines and heart [2–4]. The SLCO2B1 transporter modulates the levels of endogenous substrates including dehydroepiandrosterone-3-sulfate, estrone-3-sulfate and prostaglandin E_2_ [3–6]. In addition, it acts as a transporter of various therapeutic drugs including montelukast, cyclosporine, fexofenadine, and celiprolol [7–10].

Several polymorphisms have been identified in the *SLCO2B1* gene, and their frequencies are reported in different ethnic groups. Literature review shows that Nozawa et al. [11] first reported the allele frequencies of *SLCO2B1* gene, i.e., SLCO2B1*2 (c.1175C > T) and SLCO2B1*3 (c.1457C > T) in a Japanese population. Although there have been subsequent studies reporting the frequencies of *SLCO2B1* polymorphisms in different populations, there is still a limited amount of information available regarding ethnic differences in *SLCO2B1* genetic polymorphisms.

Functionally, the *SLCO2B1* c.1457C > T variant was associated with a reduced activity in an in vitro study [11], and the c.601G > A polymorphism has been associated with markedly reduced transport activity in vitro [12]. Additionally, it was presented that the c.935G > A polymorphism is associated with a significant reduction of SLCO2B1 activity in vitro and in vivo [8]. Considering that membrane transporters are important modulators of drug disposition [13], it has been established that genetic polymorphisms in genes encoding these transporters may account for inter-individual variability in the pharmacokinetics and pharmacodynamics of drugs.

However, the genetics and functional consequences of *SLCO2B1* variants have not been well characterized. Therefore, we analyzed non-synonymous SNPs of the *SLCO2B1* gene using pyrosequencing methods. Previous genetic analyses of *SLCO2B1* variants were conducted either by PCR–RFLP or real-time PCR [1, 8, 11]. Pyrosequencing is a non-electrophoretic, real-time DNA sequencing technology [14]. It involves the hybridization of a primer to a single-stranded PCR template, and initiation of the sequencing analysis by addition of nucleotides. The nucleotides are added sequentially, and through coupled enzymatic reactions, the polymerase-catalyzed incorporation of nucleotides can be monitored as light peaks in a pyrogram. Pyrosequencing is consistent, easy to use, economically viable, and allows for the generation of high throughput analysis with a very high success rate [15].

In this study, we developed pyrosequencing methods that can detect non-synonymous SNPs of *SLCO2B1* polymorphisms. Using this technique, we assessed the allelic frequencies of *SLCO2B1* polymorphisms in a Korean population, and compared these allelic frequencies to those reported for other ethnic groups.

## Materials and methods

### Subjects and methods

Genomic DNA samples were obtained from 227 unrelated male and female Korean subjects and written and informed consent was obtained. The protocol for *SLCO2B1* DNA analyses was approved by the ethical committee of Anam Hospital, Korea University College of Medicine, Seoul, Korea.

### Pyrosequencing method for detection of SLCO2B1 polymorphisms

Genomic DNA was isolated from peripheral leukocytes, as described previously [16, 17]. We developed the pyrosequencing method to identify the following non-synonymous SNPs of the *SLCO2B1* gene: c.1175C > T (rs1621378), c.1457C > T (rs2306168), c.43C > T (rs56837383), c.935G > A (rs12422149) c.601G > A (rs35199625) and c.644A > T (rs72559740) (Table 1). The primers used for the PCR reaction for *SLCO2B1* genotyping and pyrosequencing are described in Table 2. PCR reactions were carried out to amplify sequences to identify each *SLCO2B1* SNP using newly developed primer sets after attaching biotin to the 5′ end of each forward (or reverse) primer using PSQ Assay Design software (Pyrosequencing AB, Uppsala, Sweden). The DNA fragments containing *SLCO2B1* polymorphic sites were amplified using newly developed primer sets after attaching biotin to the 5′ end of each forward (or reverse) primer using PSQ Assay Design software (Pyrosequencing AB, Uppsala, Sweden). PCR was performed in a reaction volume of 30 μl containing genomic DNA (30 ng), 10 × PCR buffer, dNTPs (0.25 mM), 10 pmol primers (1 μl each) and 5U Taq polymerase (iNtRON, Seongnam, Korea). PCR reactions were carried out with an initial denaturation step of 94 °C for 3 min, followed by 40 cycles of denaturation at 94 °C for 30 s, annealing at 58–63 °C for 30 s and extension at 72 °C for 30 s. A final termination step was performed at 72 °C for 5 min (Table 2).

**Table 1:** Six non-synonymous nucleotide polymorphisms in the *SLCO2B1* gene

SNP	Reference number	Position	Amino acid change
SLCO2B1*2 (c.1175C > T)	rs1621378	Exon 9	Thr392Ile
SLCO2B1*3 (c.1457C > T)	rs2306168	Exon 10	Ser486Phe
*c.935G* > *A*	rs12422149	Exon 7	Gln312Arg
c.43C > T	rs56837383	Exon 2	Pro15Ser
c.601G > A	rs35199625	Exon 5	Met201Val
c.644A > T	rs72559740	Exon 5	Val215Asp

**Table 2:** Oligonucleotide primers used for PCR and pyrosequencing to detect *SLCO2B1* polymorphisms

SNP	Primer	Sequences	Size (bp)	PCR (Tm; °C)
c.43C > T	Forward	5′-CTTGGTTCTGAGGTCTAGG-3′	166	58
	Reverse	B 5′-CCTCCAGGTGTGTTTTCT-3′		
	Sequencing	5′-GCGGGTGAGGTACCCCAG-3′		
c.601G > A	Forward	5′-AACCCAGCATCTGAGTGT-3′	145	58
	Reverse	B 5′-GTCCTCACCGAGGTAGAG-3′		
	Sequencing	5′-CACAGACCCTGCTGGGC-3′		
c.644A > T	Forward	5′-AACCCAGCATCTGAGTGT-3′	145	58
	Reverse	B 5′-GTCCTCACCGAGGTAGAG-3′		
	Sequencing	5′-TTTGGCATCTCCTACAT-3′		
c.935G > A	Forward	B 5′-CCCCTACTTCTTCTTCCC-3′	115	58
	Reverse	5′-GACATGGAGGGAGCTTAC-3′		
	Sequencing	5′-TGTGACTGCTAAGACCTTT-3′		
c.1175C > T	Forward	5′-GTAGGAGGCTGTGATGGA-3′	88	58
	Reverse	B 5′-CCAGGTATGCTTGTCATC-3′		
	Sequencing	5′-CCAGGAACTTGGGCAGG-3′		
c.1457C > T	Forward	5′- ACCCTACTGGTCTTCTCTC-3′	141	63
	Reverse	B 5′-TGGCAGGGTGTGATGTATT-3′		
	Sequencing	5′-CCCACCCTGGGCTGGA-3′		

B = biotinylated at the 5′-end of the primer

For pyrosequencing reactions, 25 μl of the PCR template in a single well was immobilized by incubation (with shaking at 1,400 rpm, 10 min, room temperature) with a mixture of 5 μl streptavidin beads (Streptavidin Sepharose™ High Performance, GE Healthcare Bio-Science AB, Sweden) and 40 μl binding buffer. For primer annealing, 40 μl annealing buffer containing 0.4 μM sequencing primer was incorporated into each well. For strand separation, all liquid was removed by a Vacuum Prep Workstation (Pyrosequencing AB, Uppsala, Sweden). The beads captured on probes were incubated in 70 % ethanol and the solution was flushed through the filters for 5 s. The beads were then treated with a denaturing solution (0.2 M NaOH) that was flushed through the filters for 5 s. A wash buffer (10 mM Tris–acetate, pH 7.6) was used to rinse the beads for 5 s. All liquid was completely drained from the probes, and then the beads were released into a PSQ 96 Plate Low (Pyrosequencing AB, Uppsala, Sweden) containing the sequencing primer. The PSQ 96 Plate Low was heated at 85 °C for 2 min, and the reactions were allowed to cool to room temperature. The resulting mixture was analyzed on a PSQ 96MA Pyrosequencer (Pyrosequencing AB, Uppsala, Sweden). The accuracy of pyrosequencing was validated by direct DNA sequencing for the randomly selected samples using the same genomic DNA.

### Statistical analysis

Genetic equilibrium and linkage disequilibrium were assessed according to the Hardy–Weinberg formula using SNPalyzer ver 7.0 (DYNACOM Co., Ltd, Yokohama, Japan).

## Results

**Fig. 1:**
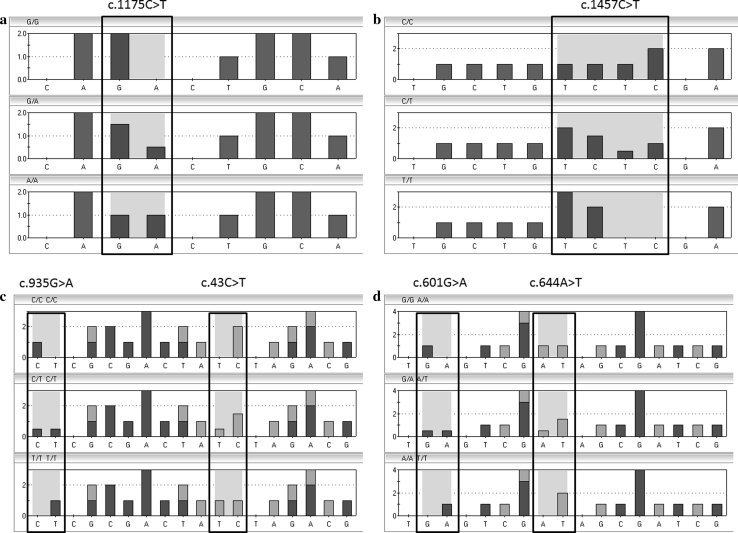
Pyrosequencing histograms for *SLCO2B1* SNPs predicted by pyrosequencing software. *Shaded areas* represent polymorphic sites to be interrogated. **a** SLCO2B1*2 (c.1175C > T), **b** SLCO2B1*3 (c.1457C > T), **c** multiplex c.43C > T/c.935G > A and **d** multiplex c.601G > A/c.644A > T

**Fig. 2:**
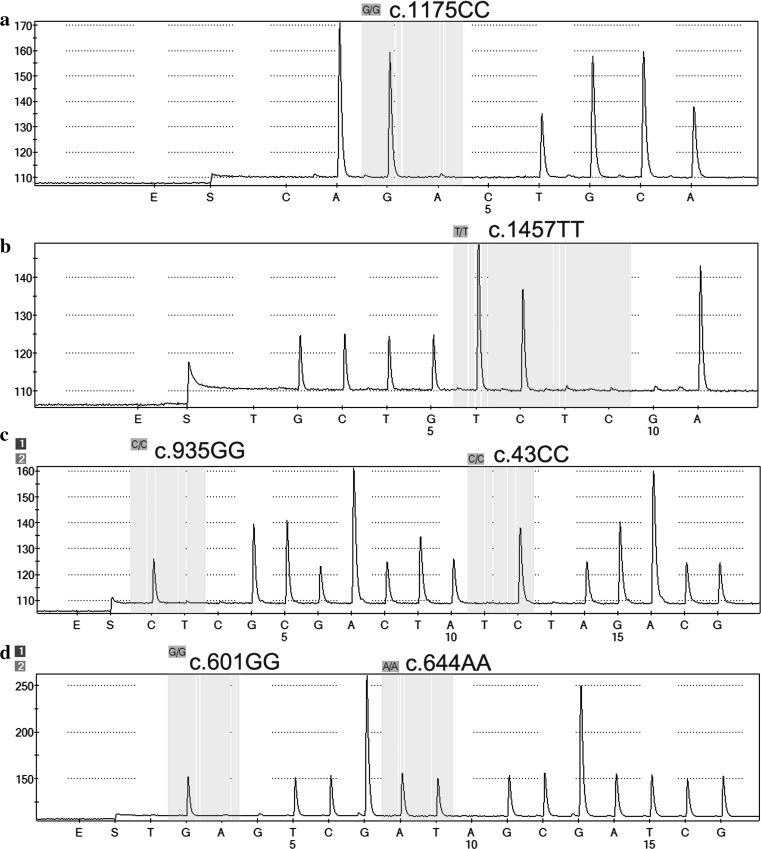
Representative pyrosequencing pyrograms for **a** SLCO2B1*2 (c.1175C > T), **b** SLCO2B1*3 (c.1457C > T), **c** multiplex c.43C > T/c.935G > A and **d** multiplex c.601G > A/c.644A > T of the *SLCO2B1* gene

We identified each SNP for c.1175C > T and c.1457C > T using a singlet pyrosequencing method, but multiplex pyrosequencing methods were applied to identify the c.43C > T and c.935G > A or c.601G > A and c.644A > T SNPs simultaneously. Representative predicted histogram patterns for each genotype are presented in Fig. 1. The assay was designed to generate a specific sequence for each SNP by setting a suitable nucleotide addition order. Nucleotide sequences and pyrograms obtained for each SNP were consistent with the predicted histograms (Fig. 2). The sequencing data obtained from the pyrosequencing method was validated by direct DNA sequencing of each SNP for randomly selected samples, and the results showed 100 % concordance with the multiplex pyrosequencing results, indicating 100 % specificity and sensitivity for the newly developed method.

When we analyzed the six non-synonymous *SLCO2B1* genetic polymorphisms with the newly developed method in 227 unrelated Korean subjects, the observed allele frequencies of *SLCO2B1* polymorphisms were as follows: 0.0 for c.1175C > T, c.43C > T and c.644A > T, 0.2687 for c.1457C > T, 0.4273 for c.933G > A, and 0.0727 for c.601G > A (Table 3). The allele frequency of c.935G > A in the population tested met Hardy–Weinberg equilibrium (χ^2^ = 0.2795, *P* = 0.597 for c.935G > A and χ^2^ = 0.0877, *P* = 0.767 for c.601G > A) whereas c.1457C > T were not in Hardy–Weinberg equilibrium (χ^2^ = 4.2559, *P* = 0.0391). When we compared the allele frequencies of *SLCO2B1* polymorphisms in this population with those in other ethnic groups, the c.1457C > T polymorphism frequency were found to be similar to those reported for a Japanese population, but differed from those of other ethnic groups including Caucasians and African-Americans (Table 4).

**Table 3:** Genotyping and allele frequencies of *SLCO2B1* polymorphisms in this study

SNP	Genotype	No.	Frequencies	Allele	Frequencies
c.1175C > T	G/G	227	1.0000	G	1.000
c.1457C > T	C/C	128	0.5639	C	0.7313
	C/T	76	0.3348	T	0.2687
	T/T	23	0.1013		
c.43C > T	C/C	227	1.0000	C	1.000
c.933G > A	G/G	72	0.3172	G	0.5272
	G/A	116	0.5110	A	0.4273
	A/A	39	0.1718		
c.601G > A	G/G	195	0.8590	G	0.9272
	G/A	31	0.1336	A	0.0727
	A/A	1	0.0004		
c.644A > T	A/A	227	1.0000	A	1.000

**Table 4:** Comparisons of *SLCO2B1* allele frequencies with those in other ethnic groups

	Population	Frequency (%)	Reference
c.1175C > T	Korean (n = 227)	0.00	Present study
	Japan (n = 534)	0.00	[11]
c.1457C > T	Korean (n = 227)	26.87	Present study
	Japanese (n = 534)	30.90	[11]
	Finnish (n = 552)	2.80	[1]
c.43C > T	Korean (n = 227)	100	Present study
c.935G > A	Korean (n = 227)	42.73	Present study
	Finnish (n = 552)	13.60	[1]
	African-American (n = 20)	13.16	[8]
	Caucasian (n = 55)	8.18	[8]
c.601G > A	Korean (n = 227)	7.27	Present study
	Finnish (n = 552)	2.10	[1]
c.644A > T	Korean (n = 227)	0.00	Present study

## Discussion

In this study, we developed a rapid and robust pyrosequencing method to detect six non-synonymous *SLCO2B1* SNPs and applied this technique to identify these SNPs in a Korean population. We observed that there were substantial differences in allele frequencies of *SLCO2B1* genotypes between our Korean sample and other ethnic groups.

To our knowledge, this is the first study to identify *SLCO2B1* polymorphisms using a pyrosequencing method. Previously, *SLCO2B1* SNPs were detected through PCR–RFLP [7, 11, 18] and real-time PCR [1, 8].

SLCO2B1 is expressed in the liver, spleen, placenta, lungs, kidneys, heart, ovaries, small intestine, and brain [19]. A number of endogenous and exogenous compounds act as substrates of the SLCO2B1 transporter [6, 13]. A number of studies have shown that *SLCO2B1* SNPs could cause a functional change in SLCO2B1 transport activity. Nozawa and his colleague first reported the role of polymorphic *SLCO2B1* genes in modulating the expression levels of OATP2B1 proteins in vitro [11]. The protein expression for SLCO2B1*2 (c.1157C > T) and SCLO2B1*3 (c.1457C > T) was 71.1 and 42.5 % compared with SLCO2B1*1 (wild type), respectively [11]. Furthermore, c.1457C > T exhibited decreased transport activity in HEK cells [20]. It has consistently been reported that subjects with the c.1457TT polymorphism exhibit 36 % lower AUC values but 52 % higher oral clearance values for fexofenadine compared with those with c.1457CC [7]. Asthma patients with the SNP positioned at c.935G > A exhibited a significant reduction in plasma levels of montelukast and reduced permeability to the drug in a MDCKII cell line expressing OATP2B1 [8]. Considering these findings, we believe that genetic polymorphisms of *SLCO2B1* play a substantial role in modulation of the pharmacokinetics/pharmacodynamics of SLCO2B1 substrates in humans.

Among the genotypes we screened in this population, we did not find any genetic substitution at c.1175C > T, c.43C > T or c.644A > T. We attempted to determine whether there is an ethnic difference frequencies of these alleles, but were only able to find one study reporting the frequency of c.1175C > T. Consistent with the present study, this polymorphism was not observed in a Japanese population [11], suggesting that the frequency of the c.1157C > T polymorphism is relatively low and may play a minor role in Asian populations.

Other polymorphisms tested at c.1457C > T, c.935C > T and c.601G > A exhibited large differences in allele frequencies among ethnic groups. The frequencies of c.1457C > T and c.601G > A in a Finnish population were 2.8 and 2.1 %, respectively [1], while we observed frequencies of 26.9 and 7.3 % for these two polymorphisms, respectively, in our Korean sample. Considering the allele frequency of c.1457C > T was 30.9 % in a Japanese population [20], there seems to be large ethnic variability in the frequencies of *SLCO2B1* polymorphisms.

Intriguingly, the occurrence of the c.935G > A polymorphism was less than 15 % in Finnish (13.60 %), African-American (13.16 %) and Caucasian (8.18 %) populations, but we observed a 42.73 % allele frequency in our Korean participants. Among the six non-synonymous polymorphisms tested in this study, the c.935G > A SNP showed the highest polymorphic frequency, and this was also the case in other ethnic groups. Even though the results are controversial [21], previous studies revealed that the c.935G > A polymorphism influences the pharmacokinetics and pharmacodynamics of montelukast, a SLCO2B1 substrate, in humans. A comparison between patients with c.935GG and c.935GA exhibited substantial difference in montelukast concentrations and its efficacy [8, 22], suggests that those with 935AA might be affected more deleteriously in terms of montelukast treatment. Considering the large ethnic differences in *SLCO2B1* polymorphisms observed in the current study and the evidence showing the modulating effect of SLCO2B1 on their substrates’ plasma levels and efficacy [7, 8, 18], more studies are warranted to validate the role of genetic variation of *SLCO2B1* in the pharmacokinetics and pharmacodynamics of SLCO2B1 substrates.

## Conclusion

The developed pyrosequencing method is a rapid and reliable genotyping method to detect six non-synonymous SLCO2B1 polymorphisms. A large difference in *SLCO2B1* genetic polymorphisms was noted when comparing our Korean sample with other ethnic groups. The *SLCO2B1* polymorphisms at c.1175C > T, c.43C > T and c.644A > T were not found in our Korean population, but c.1457C > T, c.935G > A and c.601G > A exhibited higher frequencies in Koreans population than in Finnish, Caucasian and African-American populations.
